# Gem1 and ERMES Do Not Directly Affect Phosphatidylserine Transport from ER to Mitochondria or Mitochondrial Inheritance

**DOI:** 10.1111/j.1600-0854.2012.01352.x

**Published:** 2012-04-08

**Authors:** Tammy T Nguyen, Agnieszka Lewandowska, Jae-Yeon Choi, Daniel F Markgraf, Mirco Junker, Mesut Bilgin, Christer S Ejsing, Dennis R Voelker, Tom A Rapoport, Janet M Shaw

**Affiliations:** 1 Department of Biochemistry, University of Utah School of MedicineSalt Lake City, UT, 84112, USA; 2 Department of Medicine, National Jewish HealthDenver, CO, 80206, USA; 3 Department of Cell Biology, Howard Hughes Medical Institute, Harvard Medical SchoolBoston, MA, 02115, USA; 4 Department of Biochemistry and Molecular Biology, University of Southern DenmarkDK-5230, Odense, Denmark

**Keywords:** ERMES, ER-mitochondria, Gem1 GTPase, Mdm10, Mdm12, Mdm34, membrane contact site (MCS), mitochondrial movement and inheritance, mitochondrial phospholipids, Mmm1, yeast Miro

## Abstract

In yeast, a protein complex termed the ER-Mitochondria Encounter Structure (ERMES) tethers mitochondria to the endoplasmic reticulum. ERMES proteins are implicated in a variety of cellular functions including phospholipid synthesis, mitochondrial protein import, mitochondrial attachment to actin, polarized mitochondrial movement into daughter cells during division, and maintenance of mitochondrial DNA (mtDNA). The mitochondrial-anchored Gem1 GTPase has been proposed to regulate ERMES functions. Here, we show that ERMES and Gem1 have no direct role in the transport of phosphatidylserine (PS) from the ER to mitochondria during the synthesis of phosphatidylethanolamine (PE), as PS to PE conversion is not affected in ERMES or *gem1* mutants. In addition, we report that mitochondrial inheritance defects in ERMES mutants are a secondary consequence of mitochondrial morphology defects, arguing against a primary role for ERMES in mitochondrial association with actin and mitochondrial movement. Finally, we show that ERMES complexes are long-lived, and do not depend on the presence of Gem1. Our findings suggest that the ERMES complex may have primarily a structural role in maintaining mitochondrial morphology.

The endoplasmic reticulum (ER) forms membrane contact sites (MCS) with several different organelles, including mitochondria, peroxisomes, the Golgi apparatus, endosomes and the plasma membrane 1. These sites play critical roles in cell signaling, lipid homeostasis and apoptosis 2,3. Recent studies show that contact sites play a structural role in maintaining the normal morphology of the interacting organelles. In addition, ER-mitochondrial contact sites can be maintained as organelles move within a mammalian cell 4.

Several different proteins have been implicated in tethering the ER to mitochondria in mammals and budding yeast 2,5. One of the best-characterized tethers is the ER Mitochondria Encounter Structure (ERMES), which links the ER to the outer mitochondrial membrane in *Saccharomyces cerevisiae* 6. ERMES is composed of four proteins that associate to form a complex. Mmm1 is anchored in the ER and Mdm10 in the outer mitochondrial membrane 6,7, whereas the cytosolic protein Mdm12 and the mitochondrial-associated protein Mdm34 bridge the interaction between Mmm1 and Mdm10 in the complex 8,9. Using fluorescent markers, ERMES appears as multiple discrete puncta 8,9,10 at sites of ER-mitochondrial juxtaposition 6, suggesting that these four proteins assemble into higher order structures. In the absence of any single ERMES protein, the complex falls apart and puncta are no longer visible 6; while the morphology of the ER remains intact, the normally tubular mitochondrial network is converted into one or more large spheres 7,9,11,12.

The functional role of the ERMES complex is unclear. Mdm10 and Mdm12 were originally proposed to control mitochondrial shape and mitochondrial inheritance during cell division 7,11. Additional studies suggested that Mmm1, Mdm10 and Mdm12 attach mitochondria to the actin cytoskeleton for polarized transport to the growing yeast bud 8,13. The fact that ERMES mutants lose mitochondrial DNA (mtDNA) led to the suggestion that ERMES proteins control mitochondrial genome maintenance 9,10. Consistent with this idea, Mmm1 has been shown to co-localize with replicating mtDNA nucleoids 14. In addition, a link between actin filaments, ERMES proteins and mtDNA nucleoids appears to couple inheritance of mitochondrial membranes and genomes during division 8. Mdm10, Mmm1 and Mdm12 were also shown to function in the import and assembly of mitochondrial ß-barrel proteins 15,16,17. Finally, the ERMES complex has recently been linked to the transport of phosphatidylserine (PS) from the ER into mitochondria 6,18, where it can serve as a substrate for phosphatidylethanolamine (PE) synthesis 19,20. A role for ERMES in lipid transport is also supported by the fact that three ERMES proteins (Mmm1, Mdm12 and Mdm34) have SMP (synaptotagmin-like, mitochondrial and lipid-binding proteins) domains implicated in binding hydrophobic ligands, including lipids 21.

This bewildering list of potential functions of the ERMES complex raises the question of whether some of them are indirect. For example, it is conceivable that the deletion of ERMES components causes mitochondrial morphology or mitochondrial protein import defects, which in turn cause problems in other functions. Indeed, it was recently shown that mitochondrial morphology defects in some ERMES mutants are a secondary consequence of mitochondrial protein import defects 15.

New findings raise the possibility that ERMES is regulated by Gem1, a mitochondrial outer membrane protein in the conserved Miro (mitochondrial rho-like) GTPase family. Gem1 contains two GTPase domains and two calcium-binding EF-hand motifs, all of which are required for function 22. Correlation of *GEM1* and all four ERMES loci in a genetic interaction map suggested that these genes are functionally related 6,18. Similar phenotypes have been described for *gem1* and ERMES mutants, including abnormal mitochondrial morphology and loss of mtDNA 22. In addition, genetic studies have implicated Gem1 in mitochondrial inheritance during cell division 23 and regulation of lipid synthesis by ERMES 24. Finally, Gem1 was recently identified as a substoichiometric component of the ERMES complex 24,25.

Here, we show that the ERMES complex or Gem1 have no direct role in the transport of PS from the ER to mitochondria during the synthesis of PE. In addition, we report that mitochondrial inheritance defects in ERMES mutants are a secondary consequence of mitochondrial morphology defects, arguing against a primary role for ERMES in mitochondrial association with actin and mitochondrial movement. Finally, we show that ERMES complexes do not depend on the presence of Gem1 and are often long-lived. Taken together, our findings suggest that the ERMES complex may have primarily a structural role in maintaining mitochondrial morphology.

## Results

### The ERMES complex and Gem1 do not directly affect phosphatidylserine transport from the ER to mitochondria

The ERMES complex has been proposed to facilitate the transport of lipids between the ER and mitochondria by providing a physical link between the two organelles 6,24. Specifically, ERMES may promote the transfer of ER-synthesized PS to mitochondria. Once in mitochondria, PS is converted to PE by the phosphatidylserine decarboxylase Psd1, an enzyme located in the inner membrane (see scheme in Figure S1) 19,20. The reported genetic link between ERMES components and Gem1 raises the possibility that the Gem1 GTPase may also function in lipid transport 6,18.

**Figure 1:**
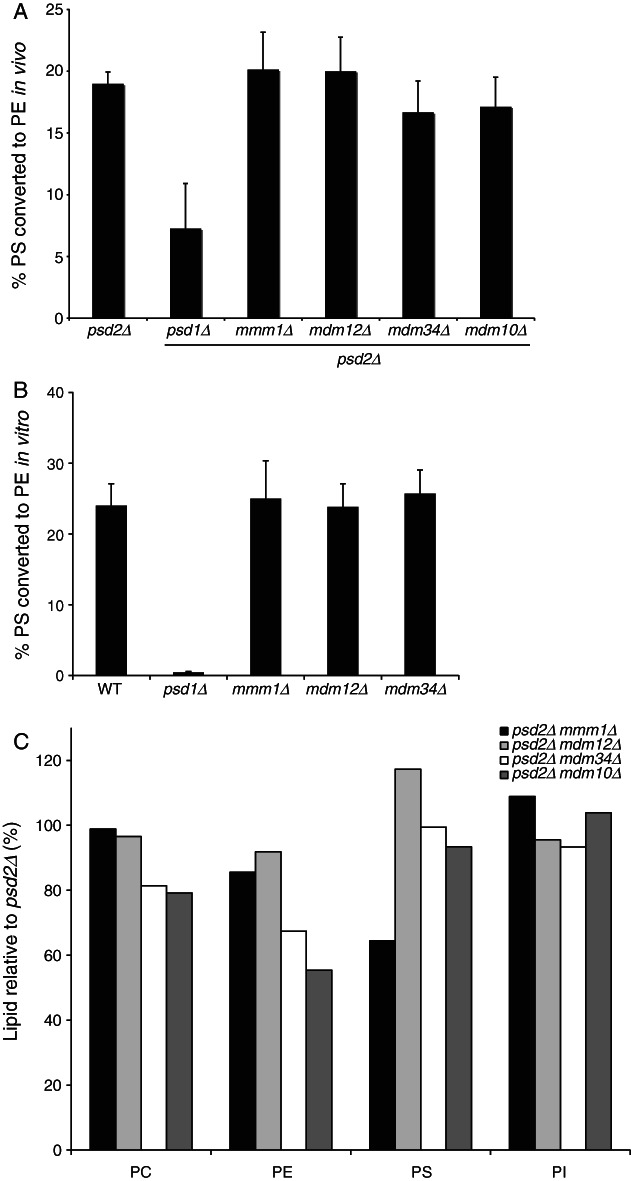
ERMES is not essential for PS transfer between the ER and mitochondria. A) The conversion of PS to PE was determined *in vivo* by incubating yeast cells with radioactive serine, extracting phospholipids, and analysis by TLC. The percentage of radiolabeled PS converted to PE in *psd2Δ* control and mutant strains is shown. Labeling of the different ERMES mutants was comparable with the exception of *mdm10Δ*, which consistently incorporated less label than *psd2Δ* control and the other mutants. B) The conversion of PS to PE was determined *in vitro* using crude mitochondria incubated with radioactive serine. The percentage of radiolabeled PS converted to PE in WT and mutant mitochondria is shown. Bars and error bars represent the average and SD from three independent experiments. C) Relative changes in phospholipid composition in ERMES mutants. Lipid extracts of the indicated strains were analyzed by shotgun lipidomics 35. The abundance of the indicated lipid classes are shown relative to the control strain *psd2*Δ. Samples were analyzed in duplicate and average values are shown. The maximal difference between two duplicate measurements was 1.2%. PS, phosphatidylserine; PE, phosphatidylethanolamine; PC, phosphatidylcholine; PI, phosphatidylinositol.

We first addressed whether ERMES is involved in PS transport from the ER to mitochondria by following the conversion of PS to PE *in vivo*. Yeast cells were incubated with radioactive serine, the phospholipids were extracted, separated by thin-layer chromatography (TLC), and analyzed by autoradiography. These studies were performed in a strain that lacks the second phosphatidylserine decarboxylase Psd2, which is localized to the Golgi and also contributes to PE synthesis. Compared with the *psd2Δ* control, the percentage of PS converted to PE is not significantly reduced in mutants that lack both an ERMES component and Psd2 (Figure 1A and S2 control). By contrast, the simultaneous absence of Psd1 and Psd2 resulted in a significant reduction (Figure 1A). The small decrease observed for *mdm34Δ* and *mdm10Δ* could result from insufficient Psd1 in the mitochondria of these strains (see Figure S3A). Similar results were obtained *in vitro*. Crude isolated mitochondria that retain ER-mitochondria connections 26 were incubated with radiolabeled serine, and the conversion of the synthesized PS to PE was determined by TLC and autoradiography. While mitochondria lacking Psd1 were totally inactive, the deletion of ERMES components had no effect (Figure 1B). Collectively, these data show that the ERMES components are not essential for the transfer of PS between the ER and mitochondria. Analysis of the total cellular phospholipid composition showed that PE and PC remained unchanged in mutants lacking both Psd2 and either Mmm1 or Mdm12, and decreased moderately in mutants lacking both Psd2 and either Mdm34 or Mdm10 (Figure 1C). PS decreased moderately in the mutant lacking both Psd2 and Mmm1, increased moderately in the mutant lacking Mdm12 and remained unchanged in the other two double mutants. These results support the conclusion that the disruption of the ERMES complex does not dramatically compromise phospholipid metabolism.

**Figure 2:**
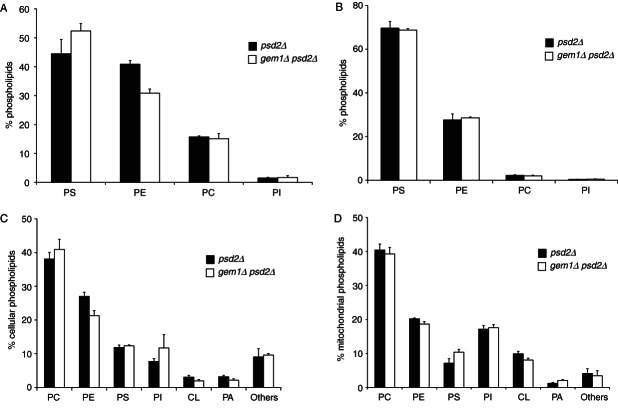
Deletion of *GEM1* does not alter cellular and mitochondrial phospholipid profiles, or the transport dependent conversion of PS to PE. A) Transport of PS and conversion into PE was assayed *in vivo* by growing the indicated strains in medium containing [^3^H]-serine and analyzing the distribution of radioactivity among phospholipid classes. (B) Synthesis and transport of PS and conversion into PE was measured *in vitro* by pulse labeling the crude mitochondrial fraction with [^3^H]-serine for 15 minutes to radiolabel the PS pool, and then arresting further PS synthesis and following the conversion to [^3^H]-PE. The reactions were terminated by lipid extraction and the distribution of radioactivity among phospholipid classes was quantified. To assess steady state lipid content, lipids were also extracted from whole cells (C), or mitochondria isolated from *psd2Δ* and *gem1Δ psd2Δ* strains (D). Individual lipid classes were separated by two-dimensional TLC and quantified by measuring phosphorus. Bars and error bars represent the average and SD from three independent experiments. CL, cardiolipin; PA, phosphatidic acid. Other lipid abbreviations as in Figure 1.

Next, we asked whether Gem1 is required for PS to PE conversion. Using the *in vivo* assay, the conversion of PS to PE was only modestly altered by the absence of Gem1 (Figure 2A). Of particular significance is the finding that no defect in PS conversion to PE was observed when experiments were performed directly on crude mitochondria *in vitro* (Figure 2B). We also compared the phospholipid composition in *psd2Δ* and *psd2Δ gem1Δ* strains. Quantification of total cellular phospholipids by TLC revealed only insignificant differences between the *gem1Δ psd2Δ* strain and a *psd2Δ* control (Figure 2C). The phospholipid compositions of mitochondrial fractions isolated from these strains were also similar (Figure 2D). These results argue against a specific role for Gem1 in regulating transport of PS into mitochondria, or a general role in phospholipid metabolism.

### The ERMES complex does not play a direct role in mitochondrial inheritance

**Figure 3:**
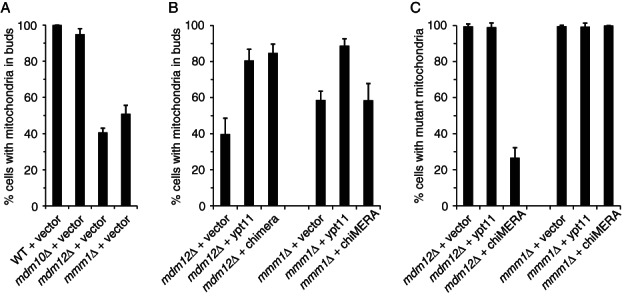
Mitochondrial inheritance defects in ERMES mutants are a secondary consequence of mitochondrial morphology defects. Quantification of mitochondrial inheritance (A) and (B) or mitochondrial morphology (C) in strains expressing a fluorescent mitochondrial marker. Genotypes of strains containing vector alone or expressing Ypt11 or ChiMERA are indicated. Bars and error bars represent the average and SD from three independent experiments.

Next, we addressed how ERMES components affect mitochondrial inheritance 7,9,11,12. During cell division in WT yeast cells, 100% of all medium- and large-sized buds inherit mitochondria from the mother cell (Figure 3A). In contrast, in *mdm12Δ* or *mmm1Δ* single mutants, only 40 or 51% of medium and large buds contain mitochondria, respectively. This is an underestimate of the defect, since we score inheritance as successful if a large bud contains only one or a few mitochondrial fragments. Although mitochondrial inheritance defects have been reported for *mdm10Δ*, in our strain background, *mdm10Δ* derived from a newly dissected tetrad displayed essentially wild-type inheritance (though it grew slowly). The *mdm10Δ* strain contained short tubular mitochondria in addition to the round or globular mitochondria characteristic of *mdm12Δ* or *mmm1Δ* (unpublished observations). Owing to its lack of mitochondrial inheritance phenotype, the *mdm10Δ* strain was not analyzed further.

To test whether the observed mitochondrial inheritance defects are a secondary consequence of mitochondrial morphology defects, we overexpressed Ypt11 in the *mmm1Δ* and *mdm12Δ* strains. Ypt11 binds to the tail of the Myo2 motor protein, and its overexpression was previously shown to promote the transport of mitochondria out of the mother cell into the bud 23,27. When overexpressed in ERMES mutants, Ypt11 increased mitochondrial inheritance from 40 to 81% in *mdm12Δ* and from 59 to 89% in *mmm1Δ* cells (Figure 3B). Importantly, overexpression of Ypt11 did not rescue mitochondrial morphology defects in the ERMES mutants; essentially 100% of *mdm12Δ* and *mmm1Δ* cells overexpressing Ypt11 retained mutant mitochondrial morphology (Figure 3C). Thus, the morphology and inheritance defects in *mdm12Δ* and *mmm1Δ* mutants are not obligatorily linked.

To further test whether morphology defects cause inheritance defects, we used an artificial tether between ER and mitochondria 28, called ChiMERA (Construct helping in Mitochondria-ER Association) 6. As reported previously 6, ChiMERA expression restored tubular mitochondrial morphology in 73% of *mdm12Δ* cells (Figure 3C). It also rescued the mitochondrial inheritance defect in this strain (Figure 3B). By contrast, ChiMERA did not rescue either the mitochondrial morphology (Figure 3B and 6) or the inheritance defects in the *mmm1Δ* mutant (Figure 3B). ChiMERA expression also did not rescue the mitochondrial morphology and inheritance defects observed in the *gem1Δ* mutant (Figure S4, and unpublished observations). When considered together, our results demonstrate that: (i) enlarged, spherical mitochondria in ERMES mutants can be transported from the mother cell to the bud during cell division, (ii) normal, tubular mitochondria are inherited more efficiently than morphologically distorted mitochondria and (iii) ERMES proteins are not essential components of the mitochondrial inheritance machinery.

**Figure 4:**
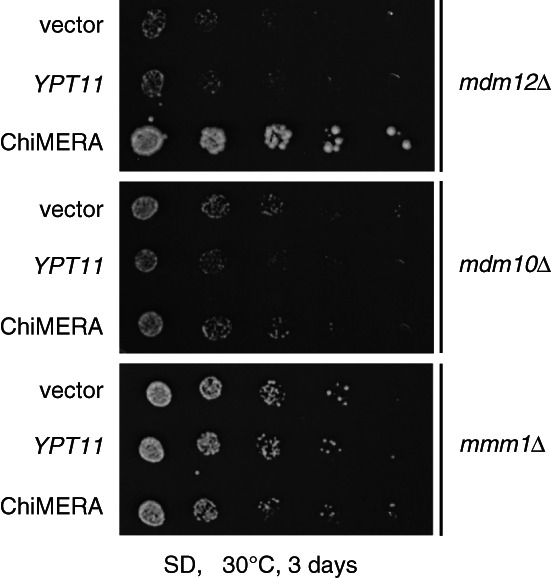
Expression of ChiMERA, but not Ypt11, rescues growth defects caused by deletion of *MDM12*. *mdm12Δ* (top) containing vector alone (vector), the synthetic ER-mitochondria tether (ChiMERA), or *YPT11* were grown on synthetic dextrose (SD) selective medium for 3 days at 30 °C. Neither ChiMERA nor *YPT11* expression rescued growth defects in *mdm10Δ* (middle) or *mmm1Δ* (bottom).

We showed previously that growth defects in *gem1Δ*, *ypt11Δ* and *mmr1Δ* mutants are correlated with mitochondrial inheritance defects 23. (Like Ypt11, Mmr1 is a Myo2 adapter that promotes mitochondrial inheritance 29.) If Gem1 acts in the same or a parallel pathway with ERMES, we would expect growth defects and inheritance defects to correlate in ERMES mutants as well. However, this was not the case. As shown previously 6, ChiMERA expression rescued the growth defect of *mdm12Δ* (Figure 4), consistent with the observation that ChiMERA rescues inheritance defects in this strain (Figure 3B). However, we found that overexpression of Ypt11 in *mdm12Δ* cells rescued mitochondrial inheritance defects, but not the growth defects (Figures 3B and 4). Thus, the growth defect of the *mdm12Δ* ERMES mutant is not caused by a mitochondrial inheritance defect. By contrast, neither ChiMERA nor Ypt11 overexpression rescued growth defects in strains lacking the integral membrane proteins Mmm1 or Mdm10 (Figure 4).

### ERMES complex formation does not require *GEM1*, *YPT11* or *MMR1*, genes implicated in mitochondrial inheritance

**Figure 5:**
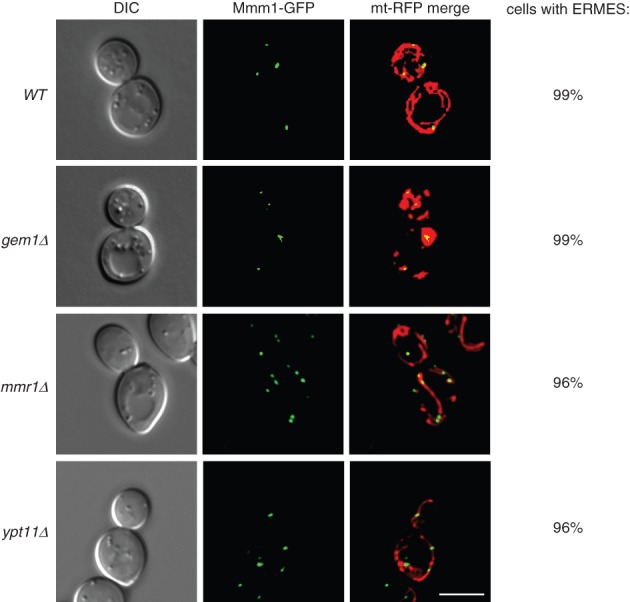
ERMES puncta formation does not require *GEM1* or genes implicated in mitochondrial inheritance. Differential interference contrast (DIC, left), Mmm1-GFP (middle), and Mmm1-GFP merged with mtRFP (right) images of the indicated strains are shown. The percentage of cells in a population containing Mmm1-GFP puncta is indicated. Bar: 5 µm.

To further test the relationship between Gem1 and ERMES, we visualized the ERMES complex by tagging its ER component Mmm1 with GFP (Mmm1-GFP). Mitochondria were simultaneously imaged using mitochondrial-targeted RFP (mtRFP). Essentially all WT cells contained multiple ERMES puncta that colocalized with the tubular mitochondrial network (Figure 5). Although previous studies reported that yeast cells contain one to ten ERMES complexes 8,9,10,24, we often observed more puncta, all of which aligned along the mitochondrial surface. Our ability to visualize more ERMES puncta per cell is likely due to differences in imaging equipment and strain backgrounds used. In addition, a subset of Mmm1-GFP marked ERMES puncta underwent rapid photobleaching, and there may be additional ERMES assemblies that are too small to visualize by fluorescence microscopy. Thus, there are likely more ERMES complexes connecting the ER to mitochondria *in vivo* than previously appreciated. Importantly, in the absence of Gem1, 99% of the cells contained ERMES puncta despite the fact that the cells have globular and fragmented mitochondria. Thus, ERMES complexes assemble at ER-mitochondrial junctions even when mitochondrial morphology is abnormal, and the aberrant mitochondrial morphology in the *gem1Δ* mutant is not due to an inability to form ERMES puncta. ERMES puncta were also observed in mitochondrial inheritance mutants *ypt11*Δ and *mmr1Δ*, which were previously shown to have synthetic growth defects with *gem1Δ* 23. As shown in Figure 5, 96% of cells in *mmr1Δ* or *ypt11Δ* strains contained ERMES puncta. It should be noted that these cells have tubular mitochondria.

**Figure 6:**
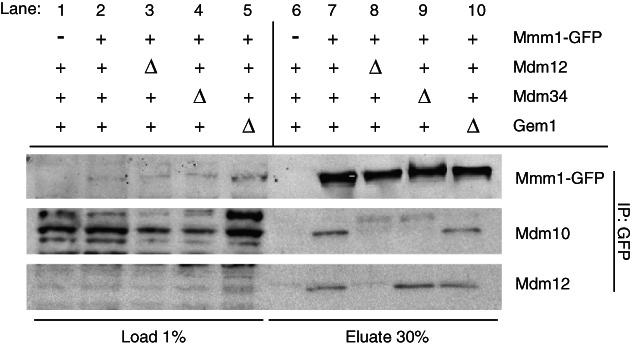
*GEM1* is not required for ERMES complex formation. Mmm1-GFP (and associated proteins) was immunopurified and the integrity of the ERMES complex analyzed by immunoblotting with antibodies against GFP or the endogenous proteins Mdm10 and Mdm12.

Immunoprecipitation experiments support the notion that Gem1 is not required for ERMES complex formation. When lysates from cells expressing functional Mmm1-GFP were subjected to immunoprecipitation with GFP antibodies, both Mdm10 and Mdm12 were co-immunoprecipitated (Figure 6, lane 7). As expected, in the absence of Mdm12 or Mdm34, Mdm10 was no longer immunoprecipitated (lanes 8 and 9). Importantly, Mdm10 and Mdm12 remain associated when Gem1 is absent (lane 10). Together, these data indicate that Gem1 is not a major structural component of ERMES and is not essential for formation of the ERMES complex. In contrast to a previous report 24, the number and appearance of ERMES puncta was normal in strains over-expressing Gem1 or in strains expressing Gem1 proteins that carry mutations in the GTPase domain or EF-hand (unpublished observations).

### The ERMES complex is a stable structure

**Figure 7:**
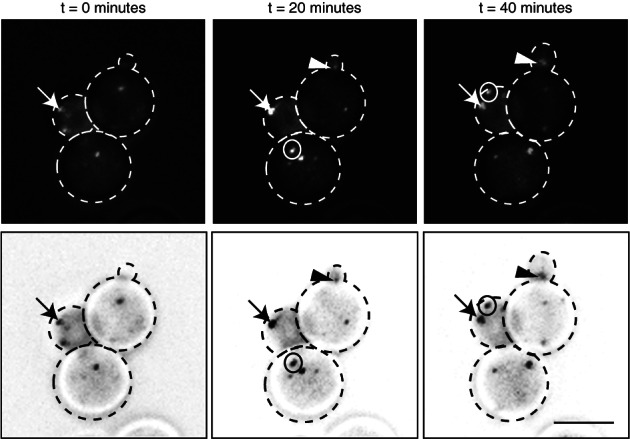
The ERMES complex is a stable structure. Dotted lines mark a cluster of three WT cells expressing the ERMES complex marker Mmm1-GFP imaged at 20 min intervals. Arrow marks a stable ERMES puncta; closed arrowhead marks a puncta moving into the growing bud; open circle marks a puncta that appears to form *de novo*. Bar: 5 µm.

Because our results suggested that the ERMES complex may be primarily involved in maintaining the structural integrity of mitochondria, we tested its stability by live-cell imaging. We used fluorescence microscopy to follow Mmm1-GFP labeled ERMES complexes over time. Over the course of 40 minutes, many ERMES puncta remained stationary within a cell (Figure 7, arrows), while some appeared to migrate (Figure 7, arrowheads mark an ERMES complex moving into the growing bud). Several examples of ERMES puncta forming *de novo* were also documented (Figure 7, open circles).

**Figure 8:**
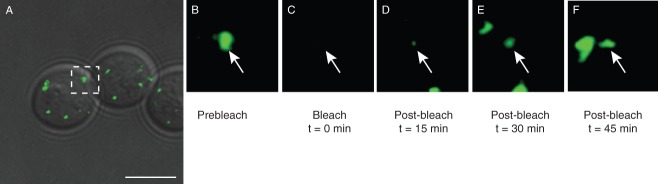
Mmm1-GFP exchanges into a stable ERMES puncta. FRAP was performed on a WT strain expressing Mmm1-GFP to mark ERMES complexes. Time-lapse images of the ERMES puncta boxed in (A) before and after photobleaching at the indicated time points. The arrow marks the position of the photobleached puncta. Bar, 5 µm.

We used fluorescence recovery after photobleaching (FRAP) to determine whether Mmm1-GFP could be incorporated into an existing ERMES puncta. As shown in Figure 8, we detected recovery of Mmm1-GFP fluorescence 15 minutes after photobleaching (compare C and D). Additional fluorescence recovery was observed 30 minutes and 45 minutes after photobleaching (Figure 8E and F). Thus, a mobile fraction of Mmm1-GFP is able to repopulate ERMES after photobleaching. No recovery of Mmm1-GFP was observed with time periods shorter than 15 minutes, indicating that the rate of recovery is extremely slow and that ERMES structures are generally long lived. Together, these results are consistent with the idea that stable ERMES complexes have a structural role at ER-mitochondrial contact sites.

## Discussion

In this report, we provide evidence that the ERMES complex and Gem1 do not directly affect lipid transport 6,24 or mitochondrial inheritance 7,8,11,12,23 as previously suggested. In addition, ERMES and Gem1 are likely functioning in distinct pathways, despite their genetic linkage and physical association 6,24,25. Our results are more consistent with a model in which the ERMES complex serves a structural role in maintaining the morphological integrity of mitochondria.

We found that the absence of ERMES components has little or no effect on the conversion of PS to PE. Thus, there is no dramatic defect in the transport of PS from the ER into mitochondria, and the small reduction in PS to PE conversion can be explained by an indirect effect of ERMES components on the import of Psd1 into mitochondria. The absence of Gem1 had no significant effect on PS to PE conversion. Consistent with our conclusions, the cellular phospholipid composition changed only slightly in mutants lacking both Psd2 and either ERMES components or Gem1. Kornmann et al. 6 did not actually report on PS to PE conversion, but they found a moderate decrease in the conversion of PS to PC in ERMES mutants. In our experiments, ERMES mutations did not affect the incorporation of radioactivity into PC (Figure S3A). In addition, minor radiolabeling of PC can occur by other pathways (i.e. the 1-carbon pathway), making it difficult to reach conclusions based on analysis of PC alone. It should also be noted that we were unable to restore tubular mitochondrial morphology in *gem1Δ* and ERMES mutants by adding lipid precursors, including ethanolamine, choline, lyso-PE or lyso-PC (Figure S5). Thus, mitochondrial morphology defects observed in *gem1Δ* and ERMES mutants are not simply a consequence of a deficiency of PC and PE.

The ERMES proteins Mmm1, Mdm10 and Mdm12 have been proposed to attach mitochondria to the actin cytoskeleton for polarized movement of mitochondria during cell division 8,13. Indeed, there are numerous reports in the literature that ERMES mutants produce large buds lacking mitochondria 7,9,11,12. Here, we provide evidence that mitochondrial inheritance defects in ERMES mutants are a secondary consequence of mitochondrial morphology changes. First, we showed that abnormally shaped mitochondria in ERMES mutants are transported into the bud upon overexpression of the inheritance-promoting factor Ypt11. Thus, aberrant mitochondria are able to bind and move on actin filaments in the absence of ERMES components. Second, we observed that restoration of tubular mitochondrial morphology by the artificial tether ChiMERA is correlated with the rescue of mitochondrial inheritance defects in *mdm12Δ* cells. The ChiMERA protein is able to restore ER-mitochondrial contact sites and tubular mitochondrial morphology but has no known actin-binding activity. Thus, mitochondrial shape is a more important determinant of mitochondrial distribution than the presence or absence of ERMES. Our combined findings argue against a model in which ERMES has a core function in mitochondrial association with, or movement along, the actin cytoskeleton.

ChiMERA expression was not able to rescue mitochondrial morphology defects in cells lacking Gem1 or the integral membrane proteins Mmm1 or Mdm10. Thus, Mdm10, Mmm1 and Gem1 carry out critical functions affecting mitochondrial shape, which cannot be bypassed by artificially tethering ER and mitochondria. Unambiguous determination of the primary molecular functions of Mdm10, Mmm1 and Gem1 in budding yeast is required to understand their roles in mitochondrial morphology maintenance.

Two groups have identified the Gem1 GTPase as a sub-stoichiometric component of the ERMES complex 24,25. We have not been able to co-precipitate untagged Gem1 with ERMES components, although we can detect untagged Gem1 in whole cell extracts (unpublished observations). Based on these results, it seems likely that only a small fraction of cellular Gem1 associates with assembled ERMES complexes. Consistent with this hypothesis, our fluorescence microscopy and immunoprecipitation experiments indicate that ERMES complexes persist when Gem1 is absent. Despite a recent report that loss of Gem1 decreases the number and increases the size of ERMES puncta *in vivo* 24, we did not observe significant differences in the number and size of ERMES puncta in WT and *gem1Δ* strains (Figure 5 and unpublished observations). Thus, Gem1 does not appear to regulate the assembly or maintenance of ERMES complexes. Since Gem1 contains GTPase domains and calcium binding motifs 30, it probably has a regulatory role, but its function remains to be elucidated.

Using time-lapse imaging, we showed that many ERMES puncta represent stationary entities. However, we also found mobile puncta, suggesting the existence of behaviorally distinct classes of ERMES structures. FRAP experiments demonstrated that the exchange of Mmm1-GFP into preexisting ERMES puncta is slow, indicating that many ERMES structures are long-lived. Some of the ERMES puncta photobleached rapidly and could not be imaged, and might represent a class of less stable, or structurally distinct, ERMES complexes. However, it appears that many ERMES complexes are simply static tethers between the ER and mitochondria.

Our results are most consistent with a model in which the ERMES complex serves as a physical link between ER and mitochondria, which is required for the maintenance of the normal morphology of mitochondria. In this model, ER structure would influence mitochondrial morphology through the ERMES complex. This is consistent with the observation that disruption of the ERMES complex leaves ER morphology intact, but disturbs the morphology of mitochondria. It is also supported by the recent observation that crossing points of ER tubules with mitochondria determine the sites of mitochondrial fission 31. The proposed model does not contradict the observation that individual ERMES components have additional functions, such as in the insertion of ß-barrel proteins into the outer membrane of mitochondria 15,16,32. On the other hand, the model suggests that some defects observed in ERMES mutants are simply a secondary consequence of mitochondrial morphology distortion.

## Materials and Methods

### Strains and plasmid construction

Standard methods were used for growth and analysis of yeast strains and plasmid construction 33. Yeast strains and plasmids are listed in Tables S1 and S2.

p415-ADH was generated by PCR amplification of the ADHpr using forward primer. 5^′^-CCGgagctcGGTGTACAATATGGACTT and reverse primer 5^′^-GCtctagaTGTATATGAGATAGTTGATT that was cloned into p415 using the restriction sites SacI and XbaI. The *PSD1* open reading frame was PCR amplified and cloned into p415-ADHpr, using restriction sites BamHI and XhoI.

### Phosphatidylserine conversion assay (in vivo)

For measuring PS to PE conversion *in vivo* (Figure 1A), overnight cultures were grown in SC dextrose medium containing 5 mm ethanolamine (Etn) and diluted in fresh media to resume growth. Aliquots of logarithmically grown cells (A600 of 0.3 OD) were sedimented by centrifugation and resuspended in 1 mL of fresh minimal medium, containing 5 mm Etn and 1 μCi L-[^14^C(U)]-serine. Samples were incubated for 2 h at 30°C followed by lipid extraction using the Folch procedure. In brief, cells were sedimented by centrifugation, resuspended in 330 μL of methanol and disrupted by vortex mixing with 100 μL of glass beads, for 10 min. Subsequently, 660 μL of chloroform was added and samples were centrifuged for 10 min at 10 000 x ***g*** to remove insoluble material. The organic solvent supernatant was recovered and washed once with 0.9% NaCl and the lower chloroform phase was dried under a stream of nitrogen. Lipids were resuspended in 15 μL chloroform and separated by TLC on Silica60 plates using chloroform/methanol/25% ammonium hydroxide (50/25/6 v/v/v). Labeled lipids were visualized using Phosphoimager (BioRad, PMI) and quantified using Metamorph software. Data reported are the mean ± SD for three experiments.

For measuring PS to PE conversion *in vivo* in Figure 2A, the *psd2Δ* and *psd2Δ gem1Δ* strains were grown in synthetic complete medium plus 2 mm Etn with glucose as a carbon source (SC). Cells in mid-log phase were harvested by centrifugation and washed twice by resuspension in water and recentrifugation. The cells were suspended in SC medium, at an *A*600 of 0.35 in a volume of 2 mL. Radiolabeling was initiated by adding 20 μCi L-[^3^H(G)] serine, and growth was continued at 30°C for 2 h with vigorous shaking. Labeled phospholipids were extracted as previously described 34 and the lipid classes were resolved by thin layer chromatography, and radioactivity was quantified by liquid scintillation spectrometry.

### Phosphatidylserine conversion assay (in vitro)

For reconstituted aminoglycerophospholipid synthesis and transport (Figure 1B), yeast cells were grown to early-logarithmic phase in synthetic dextrose media at 30 °C. Crude mitochondria (containing ER-mitochondrial contact sites) were purified as previously described 35. For lipid synthesis and transport, 100 µL crude mitochondria (100 µg protein) in 0.6 m mannitol, 20 mm Tris pH 7.4 and 0.6 mm MnCl_2_ were incubated with 0.1 μCi L-[^14^C(U)]-serine at 30°C. After 20 min, 40 mm unlabeled serine was added and PS synthesis was arrested by addition of 5 mm EDTA. The conversion of nascent PS to PE was subsequently followed in a 45 min incubation at 30°C. The reaction was stopped by adding 1 mL chloroform:methanol 2:1 (v/v). After shaking for 1 h, lipids were purified by extracting the organic phase, washing with 100 μL 0.9% NaCl (w/v) and drying at 65°C. Lipids were resuspended in 15 μL chloroform, loaded on thin-layer chromatography plates (Silica 60), separated in chloroform/methanol/25% ammonium hydroxide (50/25/6 v/v/v) and visualized using a phosphoimager (BioRad, PMI). Radioactive lipids were quantified using ImageJ software. Data reported are the mean ± SD for three experiments.

For the aminoglycerophospholipid synthesis and transport studies with crude mitochondria shown in Figure 2B the process was followed using L-[3-3H(G)] serine incorporation into lipids as previously described 36. Briefly, 20 μCi of L-[3-3H(G)] serine (32 Ci/mmol) and 0.5 mm MnCl_2_ were added to 100 μL crude mitochondria (100 µg protein) in buffer C (0.6 m sorbitol, 20 mm HEPES-KOH pH 7.4) and incubated at 30 °C for 15 min followed by incubation with EDTA (4 mm) for an additional 75 min. The initial incubation radiolabels the PS pool in the ER, and the subsequent incubation with EDTA arrests PS synthesis and allows for transport of the nascent PS to mitochondria for conversion to PE by Psd1. Radiolabeled aminoglycerophospholipids were isolated by lipid extraction and separated by thin layer chromatography on silica Gel H plates, using the solvents chloroform, methanol, 2-propanol, 0.25% KCl, triethylamine (30:9:25:6:18, v/v). The resolved radioactive lipids were quantified by liquid scintillation spectrometry 34. Data reported are the mean ± SD for three experiments.

### Lipidome analysis by mass spectrometry

Samples for lipidome analysis were harvested from 20 mL cultures of yeast growing exponentially in YP-rich medium containing 2% glucose, washed in water at 4 °C, and frozen immediately in liquid nitrogen. Lipid analysis was performed using a Triversa NanoMate ion source (Advion Biosciences, Inc.) and a LTQ Orbitrap XL mass spectrometer (Thermo Fisher Scientific) as previously described 37. PC and PE species were monitored in positive ion mode, and PS and PI species were monitored in negative ion mode. Lipids were quantified by intensity profiling using peak intensities of monitored lipid species normalized to the total intensity of all monitored lipid species within a FT MS scan. Samples were analyzed in duplicate.

### Phospholipid class analysis in whole cells and mitochondria

For whole cell lipid analysis (Figure 2C), strains were grown to mid-log phase at 30°C in SC dextrose medium, harvested by centrifugation, and washed twice with water. For mitochondrial lipid analysis (Figure 2D), strains were grown to early log phase at 30°C in semisynthetic lactate medium (0.3% yeast extract, 0.05% CaCl_2_2H_2_O, 0.05% NaCl, 0.09% MgCl_2_6H_2_O, 0.1% KH_2_PO_4_, 0.1% NH_4_Cl, 2% lactate and 0.05% dextrose, pH5.5) supplemented with adenine (20 mg/L), uracil (20 mg/L), l-leucine (100 mg/L), l-histidine (20 mg/L), and l-tryptophan (20 mg/L). Lipids were extracted 34 from crude mitochondria 38 and phospholipids were separated by two-dimensional thin-layer chromatography (TLC) on Silica 60 plates using chloroform/methanol/ammonium hydroxide (65/35/5 v/v/v) followed by chloroform/acetic acid/methanol/water (75/25/5/2.2 v/v/v). Lipids were visualized with iodine vapor and quantified by measuring phosphorus 39. The results are shown as the percentage of total lipid phosphorus in each phospholipid fraction. Data reported are the mean ± SD for three experiments.

### Coimmunoprecipitation assays

Membrane fractions were prepared from logarithmically grown cells (YPD, 500 OD_600_ units). Cells were collected, washed in water and resupended in lysis buffer (50 mm HEPES-KOH pH 6.8, 150 mm potassium acetate, 2 mm MgOAc, 1 mm CaCl_2_, 200 mm sorbitol, 1 mm PMSF) supplemented with protease inhibitors (Complete EDTA-free Cocktail, Roche). Samples were lysed by vortexing with glass beads for 5 × 30 s. After preclearing by centrifugation at 300 × ***g***, membranes were pelleted at 50 000 × ***g*** for 30 min and resuspended in immunoprecipitation buffer (50 mm HEPES-KOH pH 6.8, 150 mm potassium acetate, 2 mm MgOAc, 1 mm CaCl_2_, 15% glycerol). Digitonin (2%) was added and membranes were solubilized for 30 min at 4°C on a nutator. Supernatants cleared by centrifugation for 30 min at 50 000 × ***g*** were loaded on GFP-TRAP resin (ChromoTek, Planegg-Martiensried, Germany) and incubated overnight at 4°C. Samples were washed four times with IP buffer (0.1% digitonin) and bound material was eluted by addition of SDS sample buffer. Input and eluted samples were analyzed by SDS-PAGE, followed by immunoblotting using antibodies against GFP (Roche, Mannheim, Germany) or endogenous proteins. Antibodies against Mdm10 and Mdm12 were kindly provided by C. Meisinger (University of Freiburg, Germany).

### Fluorescence microscopy and live cell imaging

For time-lapse studies, cells expressing Mmm1-GFP were mounted on glass-bottom dishes coated with 0.1 mg/mL concanavalin A (ConA), and visualized with a Zeiss Axiovert 200 Imaging microscope equipped with DIC optics, epifluorescence capabilities, and a 100× (NA 1.4) oil immersion objective. Optical sections (0.9 µm) were acquired at 20 min intervals for a total of four hours using a Sensicam QE cooled CCD camera. Images were analyzed with Slidebook 4.2 software using only linear adjustments of contrast and brightness. Projection view images were assembled using Adobe Photoshop and Adobe Illustrator CS5.1.

For FRAP experiments, cells expressing Mmm1-GFP mounted in SD media containing 2% low melting point agar were visualized using a Zeiss LSM 510 ConfoCor 3 microscope with a 100×, 1.4 numerical aperture (NA) oil-immersion objective (optical sections = 0.9 µm). To obtain 100% loss of fluorescence, an Mmm1-GFP puncta defined in an oval-shaped region of interest in the equatorial plane of the cell membrane was bleached using a 488 nm laser. FRAP was monitored using low laser intensity at 15-minute intervals. Projection view images were assembled and analyzed using ImageJ, Adobe Photoshop and Adobe Illustrator CS5.1.

ERMES puncta formation was scored at 30°C in WT and mutant strains expressing integrated Mmm1-GFP grown to mid-log phase (OD_600_ 0.5–1.5) in synthetic dextrose (SD; 2%) dropout media. Mitochondria were visualized as described previously with pYX142-Su9 (aa 1–69)-GFP or p414-GPD-Su9 (aa 1–69)-RFPff 22, referred to as mitochondrial-targeted GFP (mtGFP) and RFP (mtRFP), respectively. Phenotypes were quantified in at least 100 cells in three or more independent experiments. Data reported are the mean ± standard deviation (SD). Images were acquired and processed as described previously 40.

Morphology and inheritance of mitochondria labeled with mtGFP (for Ypt11 expression and lipid supplementation studies) or mtRFP (for ChiMERA expression studies) were scored in log phase strains as described previously 23. For Ypt11 overexpression experiments, strains containing p416-*MET25-YPT11* or p416-*MET25* and the mtGFP marker were grown overnight at 30°C in SD dropout media containing 0.1 mg/mL methionine. Ypt11 expression was induced for 4 h by diluting into SD media lacking methionine and cysteine. Quantification was performed as described above.

## Supporting Information

Figure S1 Mitochondrial PE biosynthesis pathway. PA is converted to PS in the ER and is subsequently transferred to the mitochondrion. PS is decarboxylated by Psd1 to form PE. Mitochondrial PE can be transferred to the ER for PC synthesis.

Figure S2 Synthesis of radiolabeled PS and conversion to PE does not saturate within the time frame of conversion rate measurements. A) Yeast cells (BY4741) were incubated with radiolabeled serine and incorporation of the label into PS, PE and PC was followed for 4h in a continuous labeling experiment. Label incorporation continued after the 2h time point when conversion rates were measured (Figure 1A and Figure S3). B) Yeast cells (BY4741) were pulsed (3h) and chased (4h) with radiolabeled serine. The graph shows the percentage of radioactivity in PS, PE and PC during the chase phase. Samples were analyzed in duplicate and average values are shown. The maximal difference between two duplicate measurements was 4%.

Figure S3 Overexpression of Psd1 increases conversion of PS to PE in WT, ERMES-mutant, and *psd1Δ* *psd2Δ* strains. The small reduction in PS to PE conversion caused by the absence of *mdm34Δ* and *mdm10Δ* in Figure 1A might be indirect, since these ERMES components are involved in the insertion of ß-barrel proteins into the outer mitochondrial membrane, and these in turn are required for the import of proteins, such as Psd1, into the inner membrane. To test this possibility, we measured radiolabeled serine incorporation into PS (black), PE (gray) and PC (white) in the indicated strains plus/minus a second copy of the *PSD1* gene expressed from the constitutive ADH promoter. (A) Conversion of PS to PE was increased in both WT and ERMES-mutants. (B) Compromised PE synthesis in a *psd1Δ psd2Δ* strain lacking phosphatidylserine decarboxylases is rescued by Psd1 overexpression. For reasons that are unclear, these results differ from previous reports that Psd1 is not rate-limiting for PS to PE conversion 41–43. Additional studies are required to resolve this issue.

Figure S4 Chimera does not rescue mitochondrial morphology defects in *gem1Δ*. Quantification of mitochondrial morphology in WT and *gem1Δ* strains containing vector alone or a plasmid expressing ChiMERA. Mitochondria were visualized by expressing mtGFP. Bars and error bars represent the average and SD from three independent experiments.

Figure S5 Supplemental choline does not rescue mitochondrial morphology defects in *gem1Δ* or ERMES mutant strains. Merged DIC and mtGFP images are shown for the indicated strains grown in the absence (-) or presence (+) of 1mm choline. *pem1Δ* lacks the phosphatidylethanolamine methyltransferase enzyme that catalyzes the first step in PE to PC biosynthesis. Choline supplementation rescues abnormal mitochondrial morphology in *pem1Δ*, but not *gem1Δ* and ERMES mutants. Similar results for the *gem1Δ* and ERMES mutant strains were obtained with 0.5mm ethanolamine, lyso-PE or lyso-PC supplementation. Bar: 5μm.

Table S1 Yeast strains 44–46

Table S2 Plasmids 47
