# Supplementary material for: Gem1 and ERMES Do Not Directly Affect Phosphatidylserine Transport from ER to Mitochondria or Mitochondrial Inheritance
Source: Traffic. 2012 Apr 8;13(6):880–90. doi: 10.1111/j.1600-0854.2012.01352.x (PMC3648210; doi:10.1111/j.1600-0854.2012.01352.x)
Supplement: Figure S1 — Mitochondrial PE biosynthesis pathway. PA is converted to PS in the ER and is subsequently transferred to the mitochondrion. PS is decarboxylated by Psd1 to form PE. Mitochondrial PE can be transferred to the ER for PC synthesis. [file tra0013-0880-sd1.doc]

**
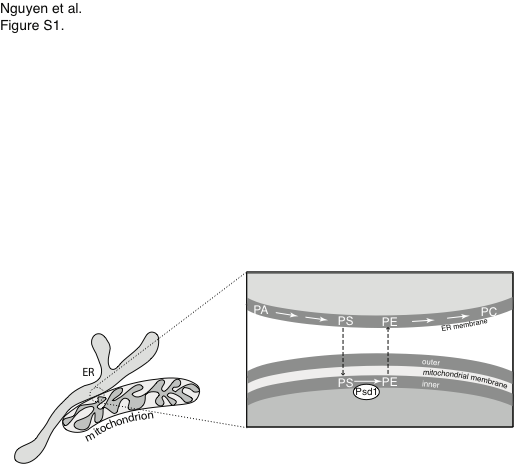
**

Figure S1: Mitochondrial PE biosynthesis pathway. PA is converted to PS in the ER and is subsequently transferred to the mitochondrion. PS is decarboxylated by Psd1 to form PE. Mitochondrial PE can be transferred to the ER for PC synthesis.
