# Supplementary material for: Gem1 and ERMES Do Not Directly Affect Phosphatidylserine Transport from ER to Mitochondria or Mitochondrial Inheritance
Source: Traffic. 2012 Apr 8;13(6):880–90. doi: 10.1111/j.1600-0854.2012.01352.x (PMC3648210; doi:10.1111/j.1600-0854.2012.01352.x)
Supplement: Figure S2 — Synthesis of radiolabeled PS and conversion to PE does not saturate within the time frame of conversion rate measurements. A) Yeast cells (BY4741) were incubated with radiolabeled serine and incorporation of the label into PS, PE and PC was followed for 4h in a continuous labeling experiment. Label incorporation continued after the 2h time point when conversion rates were measured (Figure 1A and Figure S3). B) Yeast cells (BY4741) were pulsed (3h) and chased (4h) with radiolabeled serine. The graph shows the percentage of radioactivity in PS, PE and PC during the chase phase. Samples were analyzed in duplicate and average values are shown. The maximal difference between two duplicate measurements was 4%. [file tra0013-0880-sd2.doc]

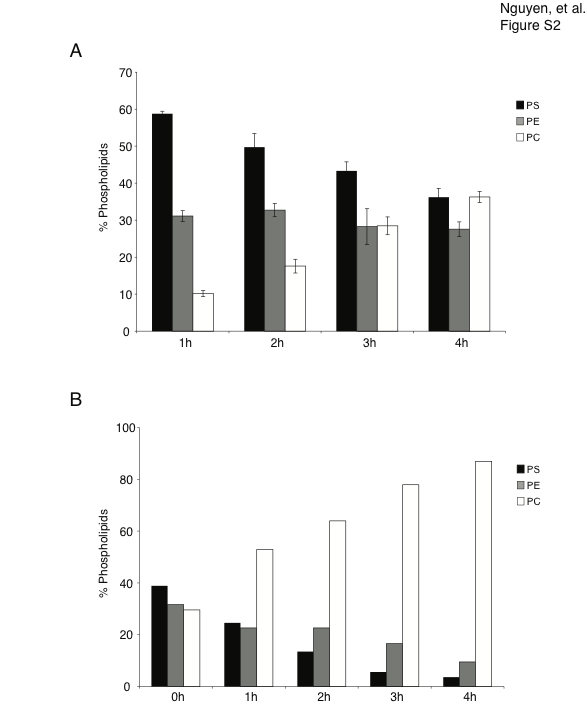


Figure S2: Synthesis of radiolabeled PS and conversion to PE does not saturate within the time frame of conversion rate measurements. A) Yeast cells (BY4741) were incubated with radiolabeled serine and incorporation of the label into PS, PE and PC was followed for 4 hours in a continuous labeling experiment. Label incorporation continued after the 2 h time point when conversion rates were measured (Figure 1A and Figure S3). B) Yeast cells (BY4741) were pulsed (3 hrs) and chased (4 hrs) with radiolabeled serine. The graph shows the percentage of radioactivity in PS, PE and PC during the chase phase. Samples were analyzed in duplicate and average values are shown. The maximal difference between two duplicate measurements was 4%.
