# Supplementary material for: Gem1 and ERMES Do Not Directly Affect Phosphatidylserine Transport from ER to Mitochondria or Mitochondrial Inheritance
Source: Traffic. 2012 Apr 8;13(6):880–90. doi: 10.1111/j.1600-0854.2012.01352.x (PMC3648210; doi:10.1111/j.1600-0854.2012.01352.x)
Supplement: Figure S3 — Overexpression of Psd1 increases conversion of PS to PE in WT, ERMES-mutant, and psd1Δ psd2Δ strains. The small reduction in PS to PE conversion caused by the absence of mdm34Δ and mdm10Δ in Figure 1A might be indirect, since these ERMES components are involved in the insertion of ß-barrel proteins into the outer mitochondrial membrane, and these in turn are required for the import of proteins, such as Psd1, into the inner membrane. To test this possibility, we measured radiolabeled serine incorporation into PS (black), PE (gray) and PC (white) in the indicated strains plus/minus a second copy of the PSD1 gene expressed from the constitutive ADH promoter. (A) Conversion of PS to PE was increased in both WT and ERMES-mutants. (B) Compromised PE synthesis in a psd1Δ psd2Δ strain lacking phosphatidylserine decarboxylases is rescued by Psd1 overexpression. For reasons that are unclear, these results differ from previous reports that Psd1 is not rate-limiting for PS to PE conversion 41–43. Additional studies are required to resolve this issue. [file tra0013-0880-sd3.doc]

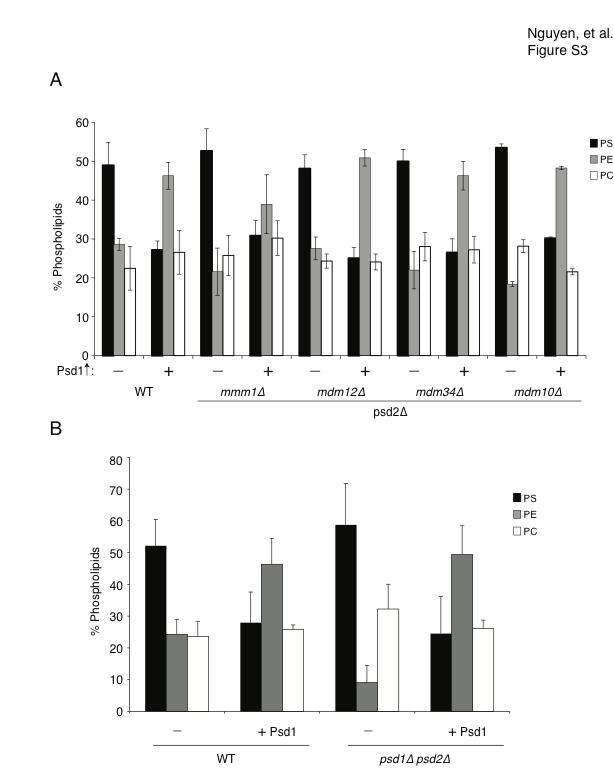


Figure S3: Overexpression of Psd1 increases conversion of PS to PE in WT, ERMES-mutant, and *psd1∆ psd2∆* strains. The small reduction in PS to PE conversion caused by the absence of *mdm34∆* and *mdm10∆* in Figure 1A might be indirect, since these ERMES components are involved in the insertion of ß-barrel proteins into the outer mitochondrial membrane, and these in turn are required for the import of proteins, such as Psd1, into the inner membrane. To test this possibility, we measured radiolabeled serine incorporation into PS (black), PE (gray), and PC (white) in the indicated strains plus/minus a second copy of the *PSD1* gene expressed from the constitutive ADH promoter. A) Conversion of PS to PE was increased in both WT and ERMES-mutants. B) Compromised PE synthesis in a *psd1∆ psd2∆* strain lacking phosphatidylserine decarboxylases is rescued by Psd1 overexpression. For reasons that are unclear, these results differ from previous reports that Psd1 is not rate-limiting for PS to PE conversion . Additional studies are required to resolve this issue.
