# Supplementary material for: Gem1 and ERMES Do Not Directly Affect Phosphatidylserine Transport from ER to Mitochondria or Mitochondrial Inheritance
Source: Traffic. 2012 Apr 8;13(6):880–90. doi: 10.1111/j.1600-0854.2012.01352.x (PMC3648210; doi:10.1111/j.1600-0854.2012.01352.x)
Supplement: Figure S4 — Chimera does not rescue mitochondrial morphology defects in gem1Δ. Quantification of mitochondrial morphology in WT and gem1Δ strains containing vector alone or a plasmid expressing ChiMERA. Mitochondria were visualized by expressing mtGFP. Bars and error bars represent the average and SD from three independent experiments. [file tra0013-0880-sd4.doc]

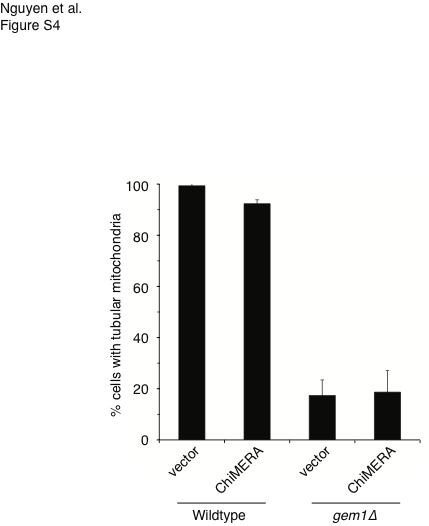


Figure S4: Chimera does not rescue mitochondrial morphology defects in *gem1∆*. Quantification of mitochondrial morphology in WT and *gem1∆* strains containing vector alone or a plasmid expressing ChiMERA. Mitochondria were visualized by expressing mtGFP. Bars and error bars represent the average and SD from three independent experiments.
