# Supplementary material for: Gem1 and ERMES Do Not Directly Affect Phosphatidylserine Transport from ER to Mitochondria or Mitochondrial Inheritance
Source: Traffic. 2012 Apr 8;13(6):880–90. doi: 10.1111/j.1600-0854.2012.01352.x (PMC3648210; doi:10.1111/j.1600-0854.2012.01352.x)
Supplement: Figure S5 — Supplemental choline does not rescue mitochondrial morphology defects in gem1Δ or ERMES mutant strains. Merged DIC and mtGFP images are shown for the indicated strains grown in the absence (-) or presence (+) of 1mm choline. pem1Δ lacks the phosphatidylethanolamine methyltransferase enzyme that catalyzes the first step in PE to PC biosynthesis. Choline supplementation rescues abnormal mitochondrial morphology in pem1Δ, but not gem1Δ and ERMES mutants. Similar results for the gem1Δ and ERMES mutant strains were obtained with 0.5mm ethanolamine, lyso-PE or lyso-PC supplementation. Bar: 5μm. [file tra0013-0880-sd5.doc]

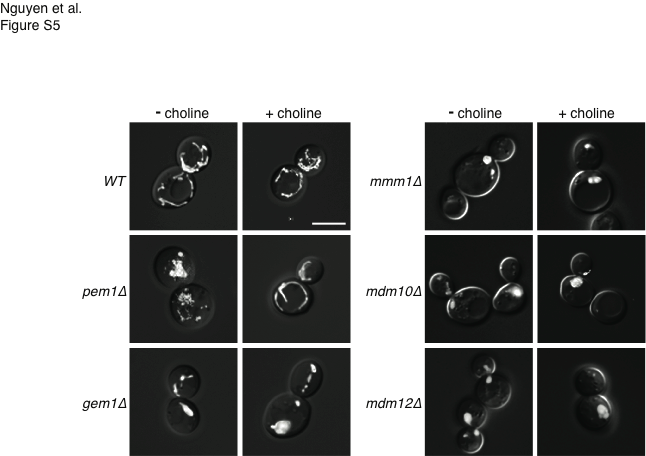


Figure S5: Supplemental choline does not rescue mitochondrial morphology defects in *gem1∆* or ERMES mutant strains. Merged DIC and mtGFP images are shown for the indicated strains grown in the absence (-) or presence (+) of 1mM choline. *pem1Δ* lacks the phosphatidylethanolamine methyltransferase enzyme that catalyzes the first step in PE to PC biosynthesis. Choline supplementation rescues abnormal mitochondrial morphology in *pem1∆,* but not *gem1Δ* and ERMES mutants. Similar results for the *gem1∆* and ERMES mutant strains were obtained with 0.5mM ethanolamine, Lyso-PE or Lyso-PC supplementation. Bar, 5 µm.
