# Supplementary material for: Gem1 and ERMES Do Not Directly Affect Phosphatidylserine Transport from ER to Mitochondria or Mitochondrial Inheritance
Source: Traffic. 2012 Apr 8;13(6):880–90. doi: 10.1111/j.1600-0854.2012.01352.x (PMC3648210; doi:10.1111/j.1600-0854.2012.01352.x)
Supplement: Table S1 — Yeast strains 44–46 [file tra0013-0880-sd6.doc]

**Table S1**

Yeast strains

| Strain ID | Mating type | Genotype | Source |
| --- | --- | --- | --- |
| JSY7000 | *MAT****a*** | *ade2-1 leu2-3 his3-11,15 trp1-1 ura3-1 can1-100* |  |
| JSY7002 | *MAT****a*** | *ade2-1 leu2-3 his3-11,15 trp1-1 ura3-1 can1-100 gem1::HIS3* |  |
| JSY9706 | *MAT****a*** | *ade2-1 leu2-3 his3-11,15 trp1-1 ura3-1 can1-100 mmm1::MMM1-GFP* | This study |
| JSY9707 | *MAT****a*** | *ade2-1 leu2-3 his3-11,15 trp1-1 ura3-1 can1-100 mmm1::MMM1-GFP gem1::HIS3* | This study |
| JSY9971 | *MAT****a*** | *ade2-1 leu2-3 his3-11,15 trp1-1 ura3-1 can1-100 mmm1::MMM1-GFP mmr1::KanMX* | This study |
| JSY9973 | *MAT****a*** | *ade2-1 leu2-3 his3-11,15 trp1-1 ura3-1 can1-100 mmm1::MMM1-GFP ypt11::KanMX* | This study |
| JSY9718 | *MAT****a*** | *ade2-1 leu2-3 his3-11,15 trp1-1 ura3-1 can1-100 mmm1::KanMX* | This study |
| JSY9722 | *MAT****a*** | *ade2-1 leu2-3 his3-11,15 trp1-1 ura3-1 can1-100 mdm10::KanMX* | This study |
| JSY9726 | *MAT****a*** | *ade2-1 leu2-3 his3-11,15 trp1-1 ura3-1 can1-100 mdm12::KanMX* | This study |
| JSY9743 | *MAT****a*** | *ade2-1 leu2-3 his3-11,15 trp1-1 ura3-1 can1-100 gem1::HIS3 psd2::KanMX* | This study |
| JSY9750 | *MAT****a*** | *ade2-1 leu2-3 his3-11,15 trp1-1 ura3-1 can1-100 psd2::KanMX* | This study |
| BY4741 | *MAT****a*** | *his3∆1 leu2∆0 met15∆0 ura3∆0* |  |
| DMY148 | *MAT****a*** | *his3∆1 leu2∆0 met15∆0 ura3∆0 MMM1::GFP-HIS3* | This study |
| DMY149 | *MAT****a*** | *his3∆1 leu2∆0 met15∆0 ura3∆0 MMM1::GFP-HIS3 mdm12::KanMX* | This study |
| DMY150 | *MAT****a*** | *his3∆1 leu2∆0 met15∆0 ura3∆0 MMM1::GFP-HIS3 mdm34::KanMX* | This study |
| DMY152 | *MAT*α | *leu2-3,112 trp1-1 can1-100 ura3-1 ade2-1 his3-11,15 gem1::KanMX MMM1::GFP-HIS3* | This study |
| DMY155 | *MAT****a*** | *his3∆1 leu2∆0 met15∆0 lys2∆0 ura3∆0* *psd1::KanMX psd2::URA3* |  |
| DMY179 | *MAT****a*** | *his3∆1 leu2∆0 met15∆0 ura3∆0 mmm1::KanMX psd2::URA3* | This study |
| DMY184 | *MAT****a*** | *his3∆1 leu2∆0 met15∆0 ura3∆0 mdm12::KanMX psd2::URA3* | This study |
| DMY185 | *MAT****a*** | *his3∆1 leu2∆0 met15∆0 ura3∆0 mdm34::KanMX psd2::URA3* | This study |
| DMY186 | *MAT*α | *his3∆1 leu2∆0 met15∆0 ura3∆0 psd2::URA3 mdm10::KanMX* | This study |
