# Supplementary material for: Gem1 and ERMES Do Not Directly Affect Phosphatidylserine Transport from ER to Mitochondria or Mitochondrial Inheritance
Source: Traffic. 2012 Apr 8;13(6):880–90. doi: 10.1111/j.1600-0854.2012.01352.x (PMC3648210; doi:10.1111/j.1600-0854.2012.01352.x)
Supplement: Table S2 — Plasmids 47 [file tra0013-0880-sd7.doc]

**Table S2**

Plasmids

| ID | Name | Purpose | Reference |
| --- | --- | --- | --- |
| B494 | p416-*MET25* | Control for *YPT11* overexpression |  |
| B1220 | pYX142-Su9(1-69)-GFP1 | Mitochondrial targeted GFP |  |
| B1641 | pYX142-Su9(1-69)-RFP1 | Mitochondrial targeted RFP |  |
| B1643 | p416-GPD-Su9(1-69)-RFPff | Mitochondrial targeted RFP |  |
| B2160 | p416-*MET25*-*YPT11* | Overexpression of *YPT11* |  |
| B2960 | p415-*GPD*-*ChiMERA* | Artificially tether ER-mitochondria |  |
| 133 | p415-*ADH* | Control for *PSD1* overexpression | This study |
| 152 | p415-*ADH-PSD1* | Overexpression of *PSD1* | This study |
